# Epidemiology of Kawasaki Disease in Europe

**DOI:** 10.3389/fped.2021.673554

**Published:** 2021-05-25

**Authors:** Maryam Piram

**Affiliations:** ^1^Department of Pediatrics, Research Centre of the Sainte Justine University Hospital, Sainte Justine University Hospital, University of Montreal, Montreal, QC, Canada; ^2^Centre for Epidemiology and Population Health (CESP), University Paris-Saclay, Le Kremlin Bicêtre, France

**Keywords:** Kawasaki disease, vasculitis, epidemiology, incidence, children, Europe, coronary arterial lesions

## Abstract

**Aim of the review:** To review major epidemiological aspects of Kawasaki disease (KD) in Europe, describing demographic characteristics, revising its incidence along with time trends and geographic variations, and describing migration studies to provide clues about its etiology.

**Recent findings:** The annual incidence of KD in Europe is about 10–15 per 100,000 children under 5 years old and seems to be relatively stable over time and space. Demographic characteristics are in line with those in other countries of the world, with a higher incidence in children from Asia and possibly North African origin. All studies performed across Europe found a coherent seasonal distribution of KD onset peaking from winter to early spring. This seasonal distribution was consistent over the years and suggests a climate-related environmental trigger. The occurrence of peaks during pandemics, microbiological findings and a possible link with southerly winds support the hypothesis of an airborne infectious agent. Neither other airborne agents such as pollutants or pollens nor urbanization and industrialization seem to have major effect on the etiology.

**Conclusion:** Discrepancies in KD incidence rates across studies were due more to methodological differences, variation in definitions and awareness of the disease than a real increase in incidence. Genetic predisposition is undeniable in KD, but environmental factors seem to play a pivotal role. Several lines of evidence support a non-exclusive airborne infectious agent with a protective immune response by the host as a key factor in inducing the inflammatory cascade responsible for symptoms and complications.

## Introduction

Kawasaki disease (KD) is the main systemic vasculitis of children under 5 years old, affecting predominantly medium-size arteries, particularly coronary arteries. This acute disease, described in 1967 by Tomisaku Kawasaki, is the leading cause of acquired heart disease in childhood in developed countries (1). The disease is described in all continents but with variable frequency. The highest annual incidence is in Asian countries, and the latest published rate (per 100,000 children under 5 years old) seems to progressively decrease from 359 in Japan, 197 in South Korea, and 95 in Shanghai to 75 in Taiwan (2–5). In non-Asian countries, the annual incidence is about 10–20 per 100,000 children under 5 years old, as described in North America, Europe, Chile or Australia (6, 7). In Hawaii, the higher incidence rate of 32 per 100,000 compared to the rest of the United States is due to the large number of children with an Asian origin (8). Little is known about the impact of KD in Africa, South America, and the Near and Middle East.

Despite extensive research, the exact cause of KD remains unknown. Important clues can be obtained by epidemiological investigations searching for etiologic factors. Disparities in incidence between countries coupled with migration studies showing a higher incidence for children with an Asian origin than Caucasian children support a genetic predisposition to the disease (8, 9). Moreover, genome-wide association studies have described polymorphisms associated with the occurrence of KD or complications (10, 11). However, genetics does not explain all of the epidemiological characteristics of the disease. Family forms are rare (2% in siblings, 1% in parents) (2). Many countries have reported an increased incidence of KD over the last decades (2–5, 12). Whether this increase is real or due to increased physician awareness is unclear. In South Korea, Shanghai and Taiwan, incidence rates seem to have stabilized in the last years (3–5). In Japan, where there is a long-standing widespread physician awareness of the disease, the increased incidence might be due to a true increase in case numbers. However, nationwide KD surveys to monitor KD every 2 years since 1970 throughout Japan showed an increased number of patients with incomplete KD without coronary artery abnormalities in the last 4 decades (2). Therefore, the increased number of patients with incomplete KD without such abnormalities could be due to better recognition of the incomplete form, a real increase of incidence or even an over-diagnosis.

Three epidemic peaks in Japan and the seasonal variation in KD support the hypothesis of an environmental factor as a trigger for KD. However, seasonal variation differs among countries. Some countries reported peaks of incidence in winter (Japan, Canada) or in spring (Taiwan) and others in summer (Korea) or autumn (India, Costa Rica) or no seasonality (Hawaii) (8, 13). Results were in agreement for seasonality in the northern hemisphere, with highest number of cases in January through March, which suggests an infectious agent operating during winter months (14). Results for a link with rainfall were discordant (13). Analyses have correlated the incidence of KD cases in Japan, Hawaii, and San Diego with tropospheric wind patterns originating from northeastern China, which suggests that a wind-borne agent, possibly a fungal toxin from agricultural sources, could trigger the illness (15). In addition, intracytoplasmic inclusion bodies found in ciliated bronchial epithelium in three dead patients suggested a viral air-borne agent (16). However, the role of a defective intestinal barrier function in the development of KD is under investigation. Increased intestinal permeability with elevated serum immunoglobulin A (IgA) level and changes in intestinal microbiota composition have been described in patients with KD (17). Age of disease onset, self-limited course of this acute disease, and low rate of recurrence suggest an environmental factor, with the host inducing a protective immune response. Bacterial, fungal and viral agents have been incriminated, but none is constantly present or universal, which suggests that non-specific agents trigger the inflammatory cascade, as observed in auto-inflammatory diseases. Other environmental agents such as toxins, food, and living standards may have modulatory effects (11).

This review aims to describe the major epidemiological aspects of KD in Europe, describing demographic characteristics, revising its incidence along with time trends and geographic variations, and describing migration studies in comparison with other geographic areas to provide clues about its etiology.

## Incidence Rates

**Figure 1:**
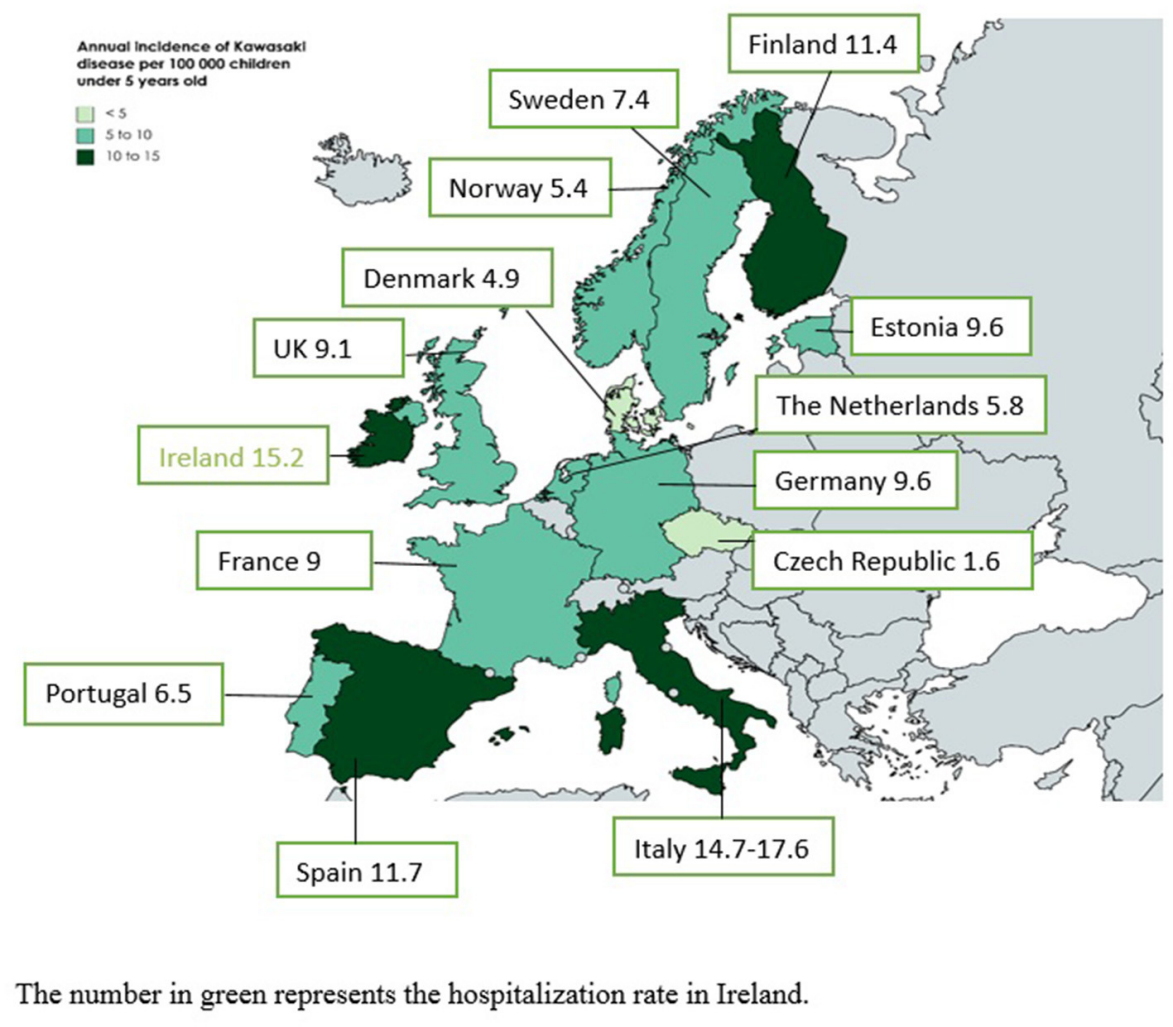
Annual incidence rates of Kawasaki disease in European countries. The number in green represents the hospitalization rate in Ireland.

Figure 1 illustrates the incidence rates of KD in European countries. Most of the studies were performed in northern and western European countries, and little is known about the epidemiology of KD in eastern European countries. The range of incidence rate is broad, with a minimum of 1.6 per 100,000 children under 5 years old in the Czech Republic and a maximum of 17.6 in Italy (18, 19). However, the period of the studies and methodological differences could explain these differences. An increased awareness of the disease in the last 20 years combined with a better definition of cases after publication in 2004 of The American Heart Association (AHA) classification criteria for KD have progressively improved the diagnosis of KD (20).

The incidence of 1.6 per 100,000 children under 5 years old in the Czech Republic comes from a study performed between 1997 and 1999, when many physicians were not aware of KD (18). Moreover, the study was prospective, with a monthly mailed questionnaire sent to heads of pediatric departments. Such voluntary reporting by busy physicians can under-record cases. The United Kingdom has the largest number of studies on the incidence of KD: annual estimates ranged from 3.4 to 15.2 per 100,000 children under 5 years old. In 1990, the incidence in the United Kingdom and Ireland was 3.4 per 100,000 children, but the period and study design were similar to the study performed in the Czech Republic and probably underestimated the true incidence (21). In 1996, using a more accurate methodology with multiple sources of case identification, Gardner-Medwin et al. found an annual incidence of 5.5 per 100,000 children in the West Midlands (England) (22). A capture–recapture analysis estimated that case finding might have missed 40% of cases. The same year in Ireland, Lynch et al. described an average annual KD hospitalization rate of 15.2 per 100,000 children (23). Because a child could be hospitalized in several hospitals or several times during the same episode, this rate was an overestimation of the true incidence. In 1998, before publication of the AHA criteria and increased awareness of KD by physicians, Harnden et al. found an incidence of 8.4 per 100,000 children by using administrative data (24). Recently, Hall et al. found an incidence of 9.1 per 100,000 children by using The Health Improvement Network database of electronic primary healthcare records from practices throughout the United Kingdom. This database has been shown to be generalizable to the UK population although with slightly fewer people aged <20 years as compared with the general UK population (25). Finally, from 2013 to 2015, Tulloh et al. found an annual incidence of 4.6 per 100,000 children with the same methodology used in 1990 and obtained a probable underestimation of the true incidence (26). The slight increase in incidence as compared with 1990 is probably due to increased awareness in the last years and because the authors contacted pediatric cardiologists in addition to pediatricians. These observations underline the importance of the methodology in interpreting results. European studies based on hospital discharge data published in the last 10 years showed annual incidence rates of 9.1–14.7 per 100 000 children under 5 years old (19, 25, 27, 28), whereas those based on prospective surveillance found lower rates (26, 29) (Table 1). A prospective surveillance design in Germany with cross validation with hospital records from two states found that the hospital-based German Pediatric Surveillance Unit (ESPED) missed 37–44% of cases. For all these reasons, the incidence of KD in Europe is probably more 10–15 per 100,000 children under 5 years old.

**Table 1:** Reported annual incidence rate (per 100,000 children under 5 years old) in the last 10 years in European countries.

**References**	**Country**	**Study period**	**Source of case identification**	**No. of cases**	**Annual incidence rate**
Cimaz et al. (19)	Italy	2008–2013	National hospital discharge record database	2,901	14.7
Riancho-Zarrabeitia et al. (27)	Spain	2005–2015	Hospital morbidity survey of the Spanish National Institute of Statistics (INE) database	3,737	11.7
Piia Jogi et al. (28)	Estonia	2008–2019	Hospital discharge records	85	9.6
Hall et al. (25)	United Kingdom	2008–2012	The Health Improvement Network (THIN) database	109	9.1
Tulloh et al. (26)	United Kingdom+ Ireland	2013–2015	Prospective surveillance	553	4.6
Tacke et al. (29)	The Netherlands	2008–2012	Prospective surveillance	341	5.8
Jakob et al. (30)	Germany	2011-2012	Prospective national surveillance (ESPED) + cross-validation with hospital record data in 2 federal states	272	7.2–9.6

## Ethnicity and Migration Studies

Similar to US studies, migration studies performed in the United Kingdom and Spain showed a higher incidence for children with an Asian origin than white peers (22, 24, 31). In England, a significant association was found between Chinese ethnicity and incidence of KD, peaking at 13.6 instead of 6.6 in areas with more than 1% of the population of Chinese ethnicity as compared with areas with low (<0.18) population of Chinese ethnicity (24). A weaker association was found between KD incidence in areas of high proportion of black ethnicity (24). In West Midlands, the incidence was 3-fold higher for Asian than white children. However, no children were Chinese, Japanese, or from other countries in the east of the Indian subcontinent but rather were of Indian, Pakistani, or Bangladeshi origin. In Spain, KD was more common in Asian and North African children (31).

## Time Trends

All studies performed across Europe found a coherent seasonal distribution of KD onset peaking from winter to early spring (24, 26, 29, 30, 32–34). This seasonal distribution was consistent over the years (24, 26, 32) and suggests a climate-related environmental trigger.

The incidence of KD seemed to remain relatively stable over time in Europe. In Denmark, the incidence progressively increased from 1980 to 1999 owing to increased awareness by physicians, with better diagnosis and an increased tendency for hospitalization, but the incidence has stabilized since 1999 (32). From 2000 to 2011, KD cases ranged from 30 to 50 per year in Portugal, with a mean of 39 cases per year (35). The annual incidence was relatively stable from 2008 to 2012 in The Netherlands, from 2008 to 2013 in Italy and from 2005 to 2015 in Spain (19, 27, 29). A study performed from 1981 to 2013 in the University hospital of Lausanne in Switzerland described an increase in number of KD cases since 1981. According to the authors, this increase resulted from better diagnosis of KD due to a better recognition of incomplete forms. Besides the increased awareness of the disease with time, the authors mentioned that the recent increase could be due to an improvement in imaging modalities with better diagnosis of cardiac complications. They also underlined that in more than 50% of patients, z-score calculations changed the degree of coronary artery involvement from the initial echocardiographic report, which had underestimated the coronary artery lesions in many cases (34). Indeed, using absolute dimension of coronary arteries could underestimate prevalence of anomalies as size of coronary arteries vary with age and body surface (36). Z-scores normalize coronary artery luminal dimension for body surface area and allow comparison across time and populations (1). However, several formulas for z-score calculation have been described yielding discrepant z-scores, which might affect diagnosis and clinical decisions (1, 36). Therefore, changes in incidence in Europe seem to be related more to increased awareness by physicians, improved imaging modalities, and modification in classification criteria (AHA and z-score calculation) than to a real increase in incidence.

## Demographic Characteristics

Although KD can occur in young adults and children of all ages, the disease has a pronounced predilection for children 1–5 years old (37–39). In Italy, the annual incidence in children under 15 years old was 5.7 per 100,000 but peaked at 14.7 in children under 5 years old (19). In a Spanish series of 625 children under 16 years old with a diagnosis of KD, 79% of cases were younger than 5, 16.5% younger than 1 and 6.7% younger than 6 months (40). The reported mean age of onset varies from 1.9 to 2.8 years, but the youngest patients could be as young as 1 month old (21, 26, 29, 30, 32–35, 40–43). Most studies, except one with very few patients in Austria, found a male predominance, with male-to-female ratios of 1.3–1.8 (19, 21, 24, 26, 29, 30, 33–35, 40–42, 44). In recent years, 58–80% of KD cases were complete as compared with 80–87% in the 1990's (21, 30, 42, 43, 45). The mean delay to treatment with intravenous Ig was 5.3–10 days (26, 29, 34, 40, 42), mostly about 7 days (21, 46, 47). Overall, 11% to 23% of children showed resistance to the first intravenous Ig infusion (29, 34, 40, 42, 46, 47). Frequencies of coronary artery lesions ranged from 2 to 65% (19, 21, 26, 34, 35, 40, 42, 46–48). This discrepancy is due to variations in definition and assessment of coronary artery involvement, time of evaluation and study design. The disease was recurrent in <2.5% of cases (34, 35, 40, 43) and mortality <0.5% in recent series (19, 26, 29, 34, 35, 40, 42, 44, 47). Familial cases are rare, reported in <1% of cases in first- and second-degree relatives (40). Appendix 1 reports the main clinical characteristics of children with KD reported in descriptive European series of more than 150 affected children.

In adulthood, KD mostly affects young adults, with a mean age at diagnosis of 31 years (range 18–68) and a slight preponderance of males (male/female ratio 1.2) (38). The disease is rare and often misdiagnosed, with a median time to diagnosis of 13 days, cardiogenic shock in 5% of cases and a high rate of coronary artery aneurysms (19%) (38).

## Geographic Variations

Seasonal variations are coherent across Europe, but geographic variations seem discordant. In Germany, KD cases were widely distributed, with no correlation among incidence rates by state and state population density per square kilometer or land under farming (30). In the United Kingdom, more cases occurred in rural than urban areas (26). Conversely, in Portugal and in The Netherlands, the incidence of KD was higher in regions with a high population density and less agriculture-based economies (29, 35). In Catalonia, Spain, most of the cases were concentrated in the most densely populated areas around Barcelona, but a significant difference was observed for the 11.5% of patients living in rural areas vs. the expected 5% for the Catalan population according to the national census. Therefore, the authors concluded that KD was more prevalent in rural than urban areas of Catalonia (31). The incidence of KD was not related to population density in Italian regions, and the authors wondered if the higher incidence in some regions might reflect better reporting (19). The same discrepancies found in Asia (15, 49, 50) suggest that urbanization and industrialization are not major etiological factors in the occurrence of KD.

## Environmental Risk Factors

An epidemiological study performed in two regions in Italy (Emilia and Romagna) found a negative correlation between temperature and number of KD cases but no correlation with precipitation or quality of air (33). Reduced onset during spring and summer seems to exclude a relation with pollen or fertilizers used in agriculture (33). The occurrence of two epidemic peaks in 2005 and 2013 in Romagna and a possible link with southerly winds and milder temperature in Emilia support the implication of an airborne infectious agent operating during winter months (33). Only 16% of Spanish patients had positive microbiological findings, but the causative agent was a respiratory virus in 35% of cases and pharyngeal Streptococcus group A in 29% (40). A tertiary pediatric center in Paris observed two peaks of hospital admission for KD between 2006 and 2020. The first peak in 2009 was concomitant with the H1N1 pandemic and the second in 2020 with the COVID-19 pandemic (51). These reports support the role of a non-exclusive upper respiratory-tract infection triggering KD. The association of social deprivation with increased incidence of KD in the United Kingdom could also support the causal role of infection but might also include other environmental determinants (24). To date, there is low evidence to incriminate exposure to other airborne agents such as pollutants or pollens (33).

## Conclusion

The annual incidence of KD in northern and western European countries is about 10–15 per 100,000 children under 5 years old and seems to be relatively stable over time and space. From the limited data available, the incidence seems to be similar in Eastern Europe. Demographic characteristics of KD in Europe are in line with those in other countries, with a higher incidence in children with Asian and possibly North African origins. Genetic predisposition is undeniable in this disease, but environmental factors appear to play a pivotal role. Several lines of evidence support that KD may have an infectious origin. Age of onset, self-limited course of the disease, coherent seasonality across Europe with more cases during winter and early spring, the occurrence of epidemic peaks during the H1N1 and COVID-19 pandemics, a possible link with southerly winds and elevated serum IgA level point to an airborne infectious agent operating during the winter months, with the host inducing a protective immune response. Overlapping features of KD with multisystem inflammatory disease in children described during the COVID-19 pandemic (52) reinforces the hypothesis of an airborne infectious trigger during KD. Studies of this newly described entity will help in understanding the pathogenic mechanisms involved in these two inflammatory diseases and possibly provide clues about host- and pathogen-dependent factors involved in the pathogenesis of KD.

## Author Contributions

MP performed the review, drafted the manuscript, and agrees to be accountable for the content of the work.

## Conflict of Interest

The author declares that the research was conducted in the absence of any commercial or financial relationships that could be construed as a potential conflict of interest.
