# Supplementary material for: Epidemiology of Kawasaki Disease in Europe
Source: Front Pediatr. 2021 May 25;9:673554. doi: 10.3389/fped.2021.673554 (PMC8185012; doi:10.3389/fped.2021.673554)
Supplement: Supplementary file 1 [file Data_Sheet_1.docx]

Appendix 1: Main clinical characteristics of children with Kawasaki disease (KD) reported in European series with more than 150 patients.

| **1^st^ author**  **(reference)** | **Country** | **Study period** | **No. of cases** | **Mean or median age at onset (range)** | **Sex ratio**  **M/F** | **Complete KD (%)** | **Median delay to treatment (range) (days)** | **Resistance to 1^st^ IVIg infusion (%)** | **CAL during acute phase (%)** | **Recur-rence**  **(%)** | **Mortality**  **(%)** |
| --- | --- | --- | --- | --- | --- | --- | --- | --- | --- | --- | --- |
| Salo (8) | Finland | 1982-1992 | 228 | 2.8 years  (7 weeks to 13 years) | 1.5 | N/A | 7 (2-20) but only 73% of patients were treated | N/A | 11 (in treated patients) | N/A | 0.9 |
| Piram (9) | France | 2011-2014 | 425 | 2.8 years  (4 weeks to 14 years) | 1.3 | 68 | 6.6 (2–38) | 22 | 39 | N/A | 0 |
| Jakob(7) | Germany | 2011-2012 | 315 | 1.9 years | 1.8 | 80 | N/A | N/A | 17 | N/A | N/A |
| Pinto(10) | Portugal | 2000-2011 | 393 | 2.8 years | 1.6 | N/A | N/A | N/A | 8.5 | 0 | 0.4 |
| Fernandez-Cooke(11) | Spain | 2011-2016 | 625 | 2.8 years  (7 weeks to 15.6 years) | 1.6 | 71 | 7.2 (0–67) | 15.7 | 23 | 2 | 0 |
| De la Harpe(12) | Switzerland | 1981-2014 | 207 | 2.7 years  (4 weeks to 15 years) | 1.4 | 71 | 7 (1–39) | 16.4 | 46 | 2.4 | 0.5 |
| Tacke(6) | The Netherlands | 2008-2012 | 319 | 2.6 years  (4 weeks to 14.6 years) | 1.5 | 78 | 7 (3–46) | 23.1 | 13.5 | N/A | 0 |
| Dhillon(13) | UK | 1990 | 163 | 2.0 years  (5 weeks to 15 years) | 1.4 | 80 | 9 (1–27) | N/A | 24 | N/A | 3.7 |
| Tulloh (5) | UK | 2013-2015 | 553 | N/A | 1.5 | 61 | 7 (without CAL)  10 (with CAL) | N/A | 24 | N/A | 0.4 |

IVIg, intravenous immunoglobulin; CAL, coronary artery lesion; N/A, non available
